# Molecular Profile and Clinical Associations of Androgen Receptor Coactivators and Structural Genes in Benign Prostatic Hyperplasia and Metabolic Syndrome

**DOI:** 10.3390/biomedicines13122896

**Published:** 2025-11-27

**Authors:** Feres Camargo Maluf, Karina Serafim da Silva, Giovana Vilas Boas Caetano, Pedro Henrique Souza Brito, Patricia Candido, Gabriel A. dos Santos, Vanessa Guimarães, Iran Amorim Silva, Alberto Azoubel Antunes, Katia Leite, Miguel Srougi, William Nahas, Ruan Pimenta, Sabrina Reis

**Affiliations:** 1 Laboratório de Investigação Médica 55 (LIM55), Faculdade de Medicina, Hospital das Clínicas HCFMUSP, Universidade de São Paulo, São Paulo 01246-903, SP, Brazil; ferescamargo@gmail.com (F.C.M.); karinasilva.09@outlook.com (K.S.d.S.); giovanacaetano07@gmail.com (G.V.B.C.); pedrohenriquesouzabrito@gmail.com (P.H.S.B.); patriciacandido11@gmail.com (P.C.); arantes_gabriel@hotmail.com (G.A.d.S.); vanessarguima@hotmail.com (V.G.); iransilva@gmail.com (I.A.S.); katiaramos@usp.br (K.L.); srougi@srougi.com.br (M.S.); sabrinareis@usp.br (S.R.); 2 Universidade Cidade de São Paulo, São Paulo 03071-000, SP, Brazil; 3 Department of Surgery, Division of Urology, Faculdade de Medicina, Universidade de São Paulo, São Paulo 01246-903, SP, Brazil; antunesuro@uol.com.br; 4 Department of Urology, D’Or Institute for Research and Education (ID’Or), São Paulo 04501-000, SP, Brazil; 5 Uro-Oncology Group, Urology Department, Institute of Cancer State of São Paulo (ICESP), São Paulo 01246-000, SP, Brazil; william.nahas@hc.fm.usp.br

**Keywords:** prostatic hyperplasia, androgen coregulators, collagen, metabolic syndrome, prostate

## Abstract

**Background/Objectives**: Benign prostatic hyperplasia (BPH) is a common condition in older men and represents a major contributor to lower urinary tract symptoms, prostate enlargement, and features of metabolic syndrome (MetS). Androgen receptor (AR) signaling and extracellular matrix (ECM) remodeling play central roles in BPH pathology, yet the clinical relevance of AR coactivators and structural genes remains incompletely understood. **Methods**: Prostate tissues from 76 BPH patients and five non-hyperplastic controls were analyzed by quantitative PCR to assess AR coactivators (*SRC-1*, *SRC-2*, *SRC-3*, *PCAF*, *p300*) and ECM-related genes (*COL1A1*, *COL3A1*). **Results**: BPH tissues showed marked overexpression of AR coactivators and collagen genes compared to controls (fold changes ≥ 7.8). Higher prostate-specific antigen (PSA) levels (≥10 ng/mL) and enlarged prostate volumes (≥100 mL) were associated with increased expression of *PCAF*, *p300*, *SRC-1*, and *COL1A1*. PSA and prostate volume correlated positively with triglycerides and VLDL, and inversely with HDL. Strong associations between collagen genes and p160 coactivators suggest coordinated androgenic and stromal remodeling activity. *COL1A1* expression was reduced in patients under pharmacological treatment, particularly with alpha-blockers or combination therapies. *PCAF* and *p300* were elevated in patients with MetS, hyperlipidemia, or hyperglycemia. **Conclusions**: These findings define a molecular signature in BPH linking androgenic, metabolic, and stromal pathology. *SRC-1*, *PCAF*, *p300*, and *COL1A1* emerge as potential biomarkers and therapeutic targets, providing new insights into the molecular mechanisms of BPH progression.

## 1. Introduction

Benign prostatic hyperplasia (BPH) is a common condition of the male lower urinary tract, and its clinical impact is expected to increase due to the global rise in life expectancy [1]. Although androgens, particularly dihydrotestosterone (DHT), play a central role in BPH pathophysiology, the molecular mechanisms underlying disease progression remain poorly understood [2]. Current treatments, whether surgical or pharmacological, are often associated with significant side effects, such as sexual dysfunction and perioperative morbidity [3,4,5], highlighting the need for less invasive and more targeted therapeutic strategies.

In this context, understanding the molecular and metabolic pathways involved in BPH has become essential. The Androgen receptor (AR) is a key mediator of the DHT response, and its inhibition has been associated with symptom improvement in BPH [6]. However, for full transcriptional activity, AR depends on nuclear coactivators, including the p160 family members, *SRC*-*1*, *SRC-2*, and *SRC-3*, as well as acetyltransferase coregulators such as *p300* and *PCAF* [7,8]. These factors are linked to several essential biological processes, such as proliferation, differentiation, and hormone-mediated signaling, and their imbalance has been associated with prostate cancer [8].

Previous studies have also shown that lipid metabolism, particularly cholesterol, the primary substrate for DHT synthesis, can modulate the activity of these coactivators [9]. The presence of metabolic syndrome (MetS) and elevated cholesterol levels has been associated with worsened BPH symptoms [10,11,12], suggesting a potential crosstalk between metabolic processes and androgen signaling. Preliminary findings from our group indicate that cholesterol can enhance AR expression by modulating p160 coactivators, supporting the hypothesis of an interface between metabolic and epigenetic factors in BPH pathophysiology [13].

Moreover, posttranslational modifications, such as AR acetylation mediated by *p300* and *PCAF*, have been associated with enhanced receptor transcriptional activity and increased prostate cell proliferation [9,14,15]. Although these mechanisms are better characterized in prostate cancer, their role in BPH remains largely unexplored. In parallel, genes involved in extracellular matrix remodeling and stromal fibrosis, such as *COL1A1* and *COL1A3*, which may be regulated via the TGF-β1 pathway, may contribute to hyperplasia development [16].

Therefore, this study aims to investigate the expression of the p160, *p300*, and *PCAF* coactivators in patients with BPH and to examine their possible associations with clinical and metabolic indicators. The integrated study of hormonal, metabolic, and epigenetic parameters may provide new insights into the pathogenesis of BPH and identify possible biomarkers and new therapeutic targets.

## 2. Methods

### 2.1. Patients

Tissue samples were collected from patients with BPH who underwent surgical treatment between 2010 and 2013, with a median follow-up time of 43.75 months (Table 1 and Table S1). Procedures were performed at the Urology Department of the Hospital das Clinicas of the University of São Paulo Medical School (HCFMUSP). Surgical specimens were used for molecular analyses, and those with prostate cancer were excluded. Control tissues were collected from the healthy peripheral zone (PZ) of five patients who underwent screening of transrectal prostate biopsies. A pathologist confirmed that all samples were free from glandular hyperplasia or carcinoma. The choice of these biopsies is based on the fact that BPH typically affects the central and transitional zones of the prostate. Therefore, these control tissues were used for comparison with the BPH samples.

Clinical data were retrieved from medical records. The following variables were included: age, preoperative PSA levels (ng/mL), prostatic volume (mL), prior pharmacologic treatment for BPH, type and classification of the previous pharmacologic treatment, type of surgery performed, presence of comorbidities, and laboratory biochemical parameters. The laboratory assessments included serum levels of glucose (mg/dL), total cholesterol (mg/dL), HDL (mg/dL), triglycerides (TGL, mg/dL), VLDL (mg/dL), and LDL (mg/dL). The PSA cutoff was set at 10 ng/mL, a value commonly used in prostate cancer screening. Prostatic volume was based on the last ultrasound performed before BPH surgery, with a cutoff of 100 mL, as reported in previous studies [17]. Patients were allocated to subgroups based on their clinical-pathological data for subsequent analyses. To classify patients with MetS, we used the criteria defined by the Adult Treatment Panel III of the U.S. National Cholesterol Education Program (NCEP-ATPIII) [13], including dyslipidemia, use of antihypertensive medication, and a clinical diagnosis of systemic arterial hypertension (SAH). The latter criterion was adopted due to the lack of complete blood pressure data in electronic medical records.

### 2.2. Extraction of RNA and Quantitative Real-Time Polymerase Chain Reaction

To extract RNA, we used the SV Total RNA isolation System (Promega, Madison, WI, USA) according to the manufacturer’s instructions. RNA integrity was assessed by agarose gel (0.8%) electrophoresis in three randomly selected samples to check the 28S and 18S bands (Figure S1). Complementary DNA (cDNA) from the total RNA was generated using a High-Capacity cDNA Reverse Transcription Kit (Applied Biosystems, Foster City, CA, USA). The target sequence was amplified in a 10 µL reaction mixture containing 5 µL of HOT FIREPol Probe Universal qPCR Mix (Solis BioDyne, Tartu, Estonia), 0.5 µL of TaqMan (Table S2), and 3.5 µL of nuclease-free water. B2M was used as an endogenous control for gene expression analysis of clinical specimens. Data were analyzed using DataAssist software V3.01 (Applied Biosystems, USA) [8].

### 2.3. Statistical Analysis

For descriptive analysis, we used the mean with standard deviation (SD) for continuous variables and percentages (%) for categorical variables. Graphs and statistical analysis were performed using GraphPad Prism 10.6.1 for Windows, with a significance level of *p* ≤ 0.05. To compare gene expression levels based on disease status and patient clinical characteristics, we used the Mann–Whitney test, Student’s *t*-test, analysis of variance (ANOVA), or the Kruskal–Wallis test. The Shapiro–Wilk test was used to assess the normality of the samples. Spearman’s test was used to analyze correlations between the expression levels of collagen genes and RA co-regulatory genes, and Fisher’s exact test was used to analyze contingency tables of the study cohort’s clinical characteristics.

## 3. Results

### 3.1. BPH Genetic Profile

**Figure 1:**
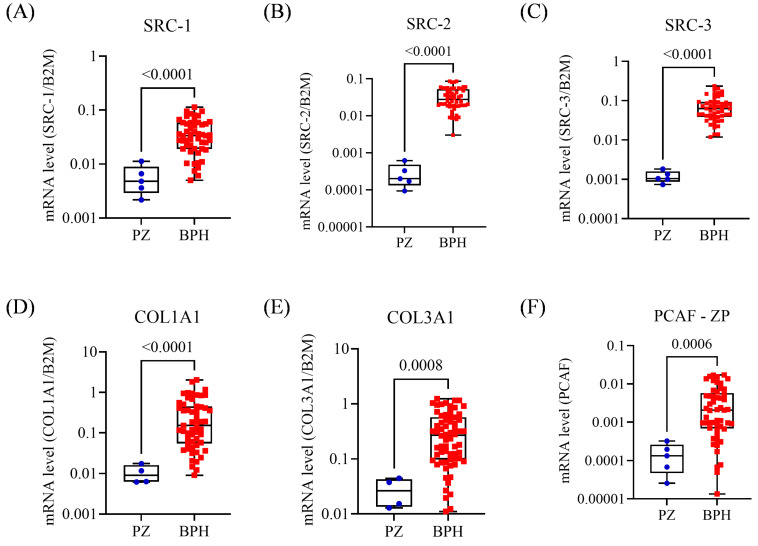
Expression profile of BPH samples. (**A**) Expression of *SRC-1* in the PZ and BPH groups, *t*-test. (**B**) Expression of *SRC-2* in the PZ and BPH groups. (**C**) Expression of *SRC-3* in the PZ and BPH groups. (**D**) Expression of *COL1A1* in the PZ and BPH groups. (**E**) Expression of *COL3A1* in the PZ and BPH groups. (**F**) Expression of *PCAF* in the PZ and BPH groups. PZ, peripheral zone; BPH, benign prostatic hyperplasia. The Mann–Whitney test was applied for all comparisons. The *p*-values obtained from the statistical analyses are shown above the bars in each panel. The error bar corresponds to the standard deviation of the samples. The circles and squares in different colors represent individual patients. The color coding was used solely to distinguish the two comparison groups (PZ and BPH) in each panel.

After statistical analysis, all genes except *p300* (Figure S2) were significantly associated with the BPH group. Compared with the healthy peripheral zones, they showed superexpression in the BPH samples (*p* < 0.005). The mean concentration levels were at least 7.8-fold higher (Figure 1A–F).

### 3.2. PSA Levels and Their Association with Gene Expression and Metabolic Parameters

**Figure 2:**
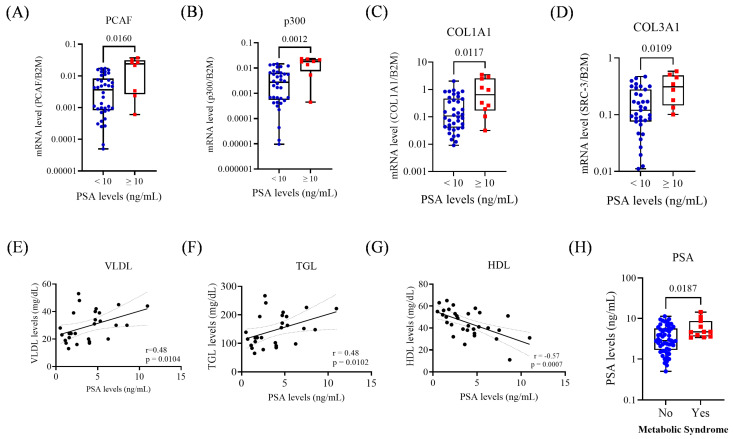
Gene expression and metabolic parameters according to PSA < 10 and ≥10 ng/mL. (**A**) Expression of *PCAF* in the PSA groups. (**B**) Expression of *p300* in the PSA groups. (**C**) Expression of *COL1A1* in the PSA groups. (**D**) Expression of *COL3A1* in the PSA groups. (**E**) Correlation between serum VLDL and PSA levels. (**F**) Correlation between serum TGL and PSA levels. (**G**) Correlation between serum HDL and PSA levels. (**H**) PSA levels and MetS status. For group comparisons in the remaining panels, statistical significance was assessed using the Mann–Whitney test. A simple linear regression analysis was performed to evaluate the association between metabolic parameters and PSA levels. The *p*-values obtained from the statistical analyses are shown above the bars in each panel. The error bar corresponds to the standard deviation of the samples.

Regarding PSA levels, the acetyltransferases PCAF and p300 were upregulated in patients with PSA ≥ 10 ng/mL, with mean expression levels 3.7 times higher than in patients with PSA < 10 ng/mL (*p* < 0.05; Figure 2A,B). Similarly, *COL1A1* and *COL3A1* were 3.9- and 1.9-fold higher, respectively, in patients with elevated PSA (*p* < 0.05; Figure 2C,D). No significant associations were observed between PSA and the p160 coactivator family genes.

Concerning metabolic markers, PSA levels showed significant positive correlations with VLDL (*r* = 0.48; *p* = 0.01) and TGL (*r* = 0.48; *p* = 0.01), and a negative correlation with HDL (*r* = −0.57; *p* < 0.0007). Additionally, patients with MetS exhibited higher PSA levels (*p* = 0.018; Figure 2E–H).

### 3.3. Prostate Volume: Associations with Gene Expression and Lipid Metabolism

**Figure 3:**
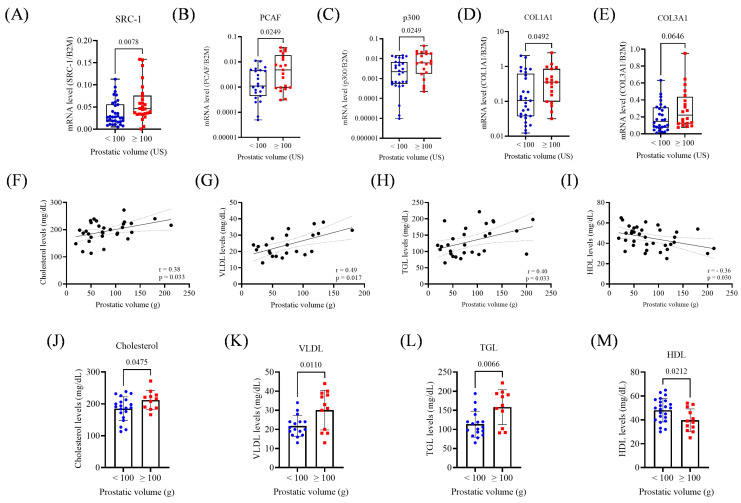
Gene expression and metabolic parameters according to prostatic volume (<100 or ≥100 mL). (**A**) Expression of SRC-1 in the prostate volume groups. (**B**) Expression of PCAF in the prostatic volume groups. (**C**) Expression of p300 in the prostatic volume groups (**D**). Expression of COL1A1 in the prostatic volume groups. (**E**) Expression of COL3A1 in prostatic volume groups. (**F**) Correlation between cholesterol levels and prostatic volume. (**G**) Correlation between VLDL levels and prostatic volume. (**H**) Correlation between TGL levels and prostatic volume. (**I**) Correlation between HDL levels and prostatic volume. (**J**) Cholesterol levels in prostatic volume groups. (**K**) VLDL levels in prostatic volume groups. (**L**) TGL levels in prostatic volume groups. (**M**) HDL levels in prostatic volume groups. US, ultrasound; g, gram. The Mann–Whitney test and unpaired *t*-test were applied depending on data distribution. A simple linear regression analysis was conducted to evaluate the association between metabolic parameters and prostatic volume. The *p*-values obtained from the statistical analyses are shown above the bars in each panel. The error bars represent the standard deviation of the samples.

Among the p160 family, only *SRC-1* was overexpressed in prostates ≥ 100 mL (1.6-fold increase; *p* < 0.05; Figure 3A). *PCAF* and *p300* were also significantly upregulated (5-fold and 2.5-fold, respectively; *p* < 0.05; Figure 3B,C). *COL1A1* showed statistically significant upregulation in the ≥100 mL group, while *COL3A1* showed a non-significant trend (*p* = 0.049; *p* = 0.064; Figure 3D,E).

Prostate volume positively correlated with total cholesterol (*r* = 0.38; *p* = 0.033), VLDL (*r* = 0.49; *p* = 0.017), and TGL (*r* = 0.40; *p* = 0.033), and negatively with HDL (*r* = −0.36; *p* = 0.030; Figure 3F–I). No correlation was found with glucose levels. Patients with prostate volumes ≥100 mL had higher serum levels of total cholesterol (*p* = 0.0475), VLDL (*p* = 0.0110), and TGL (*p* = 0.0066), and lower HDL (*p* = 0.02, Figure 3J–M).

### 3.4. Correlation Between Collagen and AR Coregulators-Positive Association with the p160 Family

After the Spearman analysis, *COL1A1* correlated with *SRC-1* (*r* = 0.436 [95% CI 0.221–0.611]; *p* < 0.0005), *SRC-2* (*r* = 0.437 [95% CI 0.217–0.615]; *p* < 0.0005), and *SRC-3* (*r* = 0.501 [95% CI 0.300–0.659]; *p* < 0.0005). A significant correlation was also established between *COL3A1* and *SRC-1* (*r* = 0.553 [95% CI 0.367–0.696]; *p* < 0.0005), *SRC-2* (*r* = 0.495 [95% CI 0.291–0.656]; *p* < 0.0005), and *SRC-3* (*r* = 0.570 [95% CI 0.390–0.708]; *p* < 0.0005) (Table 2). Both collagen genes showed no significant associations with *p300* or *PCAF*.

### 3.5. Gene Expression and Its Association with BPH Treatment and Metabolic Status

**Figure 4:**
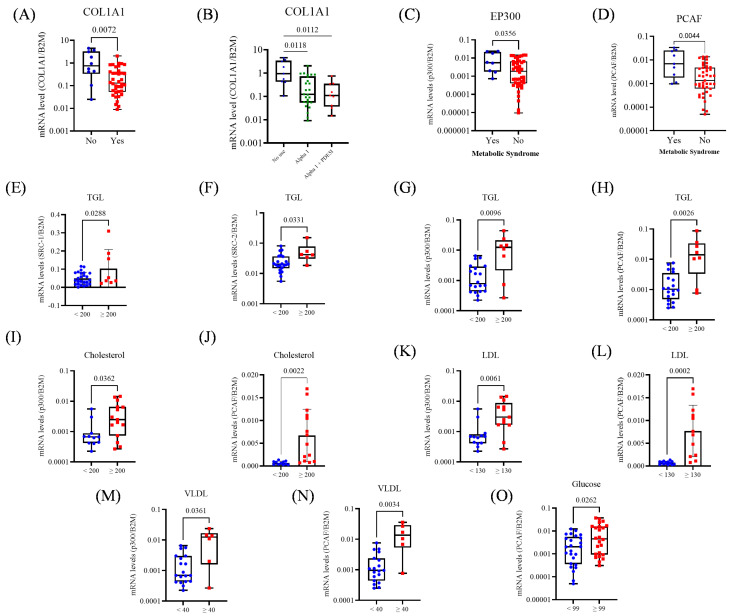
Expression of collagen genes and transcriptional coactivators in prostate tissue from BPH patients, according to pharmacologic treatment and metabolic alterations. (**A**) Comparison of *COL1A1* expression between untreated patients and those receiving pharmacologic treatment for BPH. (**B**) *COL1A1* expression according to treatment type: no therapy, alpha-1 blockers, alpha-1 blockers combined with PDE5 inhibitors. (**C**) Expression of *PCAF* and MetS status. (**D**) Expression of *p300* and MetS status. (**E**) Expression of *SRC-1* and TGL levels. (**F**) Expression of *SRC-2* and TGL levels. (**G**) Expression of *p300* and TGL levels. (**H**) Expression of *PCAF* and TGL levels. (**I**) Expression of *p300* and Cholesterol levels. (**J**) Expression of *PCAF* and Cholesterol levels. (**K**) Expression of *p300* and VLDL levels. (**L**) Expression of *PCAF* and LDL levels. (**M**) Expression of *p300* and VLDL levels. (**N**) Expression of *PCAF* and VLDL levels. (**O**) Expression of *PCAF* and Glucose levels. The Mann–Whitney test, unpaired *t*-test, and Kruskal–Wallis (Dunn’s test correction) were applied depending on data distribution. The *p*-values obtained from the statistical analyses are shown above the bars in each panel. The error bar corresponds to the standard deviation of the samples.

Patients receiving pharmacologic BPH treatment exhibited 4.8 times lower *COL1A1* expression compared to those untreated (*p* = 0.007; Figure 4A). When stratified by treatment type, patients without treatment expressed *COL1A1* at levels 4.7-fold and 8.2-fold higher than those treated with alpha-1 blockers (*p* = 0.018) or alpha-1 blockers plus PDE5 inhibitors (*p* = 0.012; Figure 4B). No significant associations were observed with *COL3A1* or coactivators. Patients with MetS showed significantly higher expression of *PCAF* (*p* = 0.004) and *p300* (*p* = 0.0356; Figure 4C,D), but no significant differences for *SRC-1*, *SRC-2*, or *SRC-3*.

Regarding metabolic parameters, elevated TGL levels (≥200) were associated with higher expression of *SRC-1* (*p* = 0.028), *SRC-2* (*p* = 0.033), *p300* (*p* = 0.009), and *PCAF* (*p* = 0.002; Figure 4E–H). Total cholesterol ≥ 200 was also associated with increased expression of *p300* (*p* = 0.03) and *PCAF* (*p* = 0.002; Figure 4I,J). Patients with LDL ≥ 130 showed upregulation of *p300* (*p* = 0.006) and *PCAF* (*p* = 0.002; Figure 4K,L), and VLDL ≥ 40 was similarly associated with elevated *p300* (*p* = 0.036) and *PCAF* (*p* = 0.003; Figure 4M,N). Hyperglycemia (glucose ≥ 99) was linked to increased *PCAF* expression (*p* = 0.026; Figure 4O).

## 4. Discussion

It is well known that age is the leading risk factor for the incidence of BPH and LUTS. Therefore, with the aging population, the prevalence of elderly BPH patients requiring treatment is rising. Consequently, most will start with pharmacologic therapy, and a significant proportion will eventually require surgical management [2,3,18,19]. However, these individuals of advanced age are more likely to have comorbidities, which have been proven to increase perioperative morbidity and mortality [20]. In addition to age itself, several commonly observed metabolic and endocrine disorders, such as insulin resistance, central obesity, changes in sex hormone levels, and low-grade chronic inflammation, have been increasingly recognized as factors contributing to the pathophysiology of BPH [21].

These interconnected processes may be related to prostatic enlargement and symptom severity. In this context, developing less invasive treatments remains relevant, and BPH genetic profiling may help identify future therapeutic avenues. In our cohort, we observed increased expression of selected genes in BPH samples and identified associations with clinical parameters, including prostate volume and PSA. Furthermore, preliminary correlations between metabolic indicators (dyslipidemia, hyperglycemia, components of MetS) and genes involved in androgen signaling and extracellular matrix remodeling suggest a potential interaction between systemic metabolic status and prostatic molecular features.

Patients with prostate volume ≥ 100 mL showed higher total cholesterol and VLDL and lower HDL. These findings are compatible with previous hypotheses linking metabolic alterations, chronic inflammation, and endothelial dysfunction to prostatic enlargement [21]. PSA levels correlated positively with VLDL and triglycerides and negatively with HDL, suggesting that lipid metabolism may contribute to PSA variability. Although other studies did not report similar findings, differences in cohort composition, especially the focus on BPH, may partially explain the discrepancy [22,23].

The association between the androgen pathway and BPH pathogenesis has been described in the literature, although the exact mechanisms remain to be elucidated. In addition, it is well known that the AR coregulators have essential roles in androgen downstream actions, including the p160 family, as well as the acetyltransferases *PCAF* and *p300* [24,25]. Our data showed overexpression of AR coregulators (except *p300*) in BPH samples relative to controls, consistent with their importance in androgen signaling. Notably, *PCAF* expression was higher in patients with altered metabolic indicators. Although *PCAF* has been associated with metabolic pathways in other tissues, its role in BPH and systemic MetS remains insufficiently explored. The observed correlations raise the hypothesis that *PCAF* may participate in the metabolic androgen interface in prostatic tissue; however, this possibility requires functional validation. [21,26].

Youn et al. established a murine BPH model to test the anti-androgenic effects of berberine, observing suppression of androgen-regulated genes, including *SRC-1* [27]. In our study, we also identified elevated *SRC-1* expression in BPH patients, particularly those with larger prostate volumes. However, like in our previous studies in PCa, no significant association was found between p160 expression and PSA, suggesting that these coregulators may not directly influence PSA levels in BPH [8].

To our knowledge, no papers have investigated the acetylators *p300* and *PCAF* in BPH. However, BPH samples are commonly used as controls for PCa studies. One example is the pre-clinical trial by Linja MJ et al., which assessed 16 AR coregulators in PCa hormone-naïve, PCa hormone-refractory, and BPH samples. Despite the small number of controls, there was no discernible difference in *p300* and *SRC-1* expression between the BPH group and the other groups [28]. The lack of difference in the analysis between the BPH group and the well-known role of AR coregulators in PCa may suggest that these coregulators also participate in BPH physiopathology. The present paper corroborates that the expression of *p300* and *PCAF* was upregulated among BPH patients with higher PSA levels and bulkier prostates. Therefore, the participation of these genes in BPH pathogenesis should be increasingly studied.

Our work shows that collagen genes were positively associated with BPH, PSA, and prostate volume. These findings are consistent with the concept that extracellular matrix remodeling accompanies BPH progression. Previous studies have demonstrated that interactions between macrophages and epithelial cells can enhance TGF-β signaling and EMT, thereby promoting collagen expression. Our findings, although not mechanistic, are compatible with these observations [24,29,30,31]. The correlations between collagen genes and p160 cofactors raise the possibility of an androgen-related regulatory axis; however, confirmatory functional studies are needed to clarify these interactions [32].

Comparisons with previous pharmacological studies in animal models revealed mixed directional effects, likely reflecting species-specific differences and treatment duration [33,34]. In our dataset, patients under combined therapy displayed lower *COL1A1* levels, although the small sample size precludes mechanistic interpretation. Therefore, while pharmacological modulation of androgen or inflammatory pathways may influence collagen expression, the present findings are insufficient to determine causality, and larger functional studies will be needed to clarify these interactions.

Prostatic baseline volume and PSA levels are established predictors of BPH progression, with larger prostates and higher PSA values associated with increased risks of acute urinary retention, surgery, and reduced treatment response [35,36,37]. In this context, our findings indicate that *SRC-1*, *PCAF*, *p300*, *COL1A1*, and *COL3A1* are associated with higher prostatic volumes or PSA levels, suggesting that these genes may serve as molecular predictors for disease progression. Further studies are warranted to validate their clinical utility.

A significant limitation of the present study is the relatively small number of control samples and the restricted power for subgroup analyses. Importantly, though, obtaining truly normal prostate tissue is inherently challenging. Despite their limited number, our control samples underwent strict histological evaluation. They represented peripheral-zone regions without hyperplastic changes, which is generally considered the closest approximation to non-hyperplastic prostate tissue available from living patients.

The use of in silico genomic datasets for experimental validation should be interpreted with caution. Although tools such as GTEx, GEPIA2, or cBioPortal are often used for exploratory comparisons, they do not include ideal controls for various diseases, including HBP. GTEx tissues come from deceased organ donors and are classified as “normal” in relation to age, meaning that mild benign hyperplasia is often considered acceptable within pathology review guidelines [38]. Similarly, TCGA PRAD contains mostly tumor-adjacent tissue rather than histologically normal prostate. These intrinsic limitations reduce the suitability of such datasets as strict controls for BPH and help explain the difficulty in integrating our findings with public data without introducing bias. Consistent with these limitations, our exploratory in silico comparisons are provided only as Supplementary Material (Figures S3–S5) and were not used for validation.

Given our results, further functional studies are needed to validate the causal relationships between the genes investigated and the clinical characteristics of BPH. Additional analyses of protein profile, duration, and treatment adherence would be essential to validate the translational relevance of our findings and to improve the interpretation of pharmacological influences on gene expression. Despite these limitations, this study is among those that integrate clinical, metabolic, and transcriptional profiles specifically in BPH. Future studies that provide more clinical information will enable a more robust, refined comparative analysis. Such efforts will ultimately deepen our understanding of the molecular determinants that underlie BPH pathophysiology. Our findings provide a descriptive overview of androgen-related coactivators and extracellular matrix genes in the context of benign prostatic disease and may help guide future mechanistic and translational investigations.

## 5. Conclusions

This study integrates clinical, metabolic, and transcriptional data to characterize molecular patterns associated with BPH. We identified correlations between metabolic dysfunction and increased expression of the epigenetic coactivators *PCAF* and *p300*, as well as between extracellular matrix genes and AR coregulators. Although these findings do not establish mechanistic links, they suggest potential interactions between metabolic status, androgen-related transcription, and tissue remodeling. Future functional studies will be required to determine whether these associations reflect causal biological pathways.

## Figures and Tables

**Table 1:** Demographic characteristics.

Characteristics	Control (*N* = 5)	BPH (*N* = 76)	*p*-Value
Age, *n* (%)	5	76	
≤65	4 (80)	27 (34.6)	0.070 ^$^
>65	1 (20)	49 (67.9)	
PSA, *n* (%)	5	61	
<10	5 (100)	50 (64.1)	0.58 ^$^
>10	0	11 (14.1)	
Prostatic Volume, *n* (%)		66	
<100		40 (51.2)	
>100		27 (34.6)	
Use of Medications, *n* (%)		66	
Yes		53 (80.3)	
No		13 (19.7)	
Use of Medications, *n* (%)		53	
Alpha Blocker		38 (71.6)	
5-alpha-reductase Inhibitor		1 (1.8)	
Anticholinergic		1 (1.8)	
Alpha Blocker + 5-alpha-reductase Inhibitor		12 (22.6)	
Alpha Blocker + Anticholinergic		2 (3.7)	
Type of Surgery		71	
Transurethral Resection of the Prostate		43 (60.5)	
Transvesical Prostatectomy		14 (19.7)	
Millin’s Prostatectomy		14 (19.7)	
Comorbidity		69	
SAH		44 (64)	
Diabetes Mellitus		13 (19)	
MetS		12 (17)	
Glucose (mg/dL)		69	
<99		50 (72)	
≥99		19 (28)	
Total Cholesterol (mg/dL)		38	
<200		20 (53)	
≥200		18 (47)	
HDL (mg/dL)		38	
<100		14 (37)	
≥100		24 (63)	
TGL (mg/dL)		37	
<200		20 (54)	
≥200		17 (46)	
VLDL (mg/dL)		34	
<100		28 (82)	
≥100		6 (18)	
LDL (mg/dL)		38	
<130		24 (63)	
≥130		14 (37)	

N, number of patients; PSA, prostate-specific antigen; SAH, systemic arterial hypertension; MetS, metabolic syndrome; HDL, high-density lipoprotein; TGL, triglycerides; VLDL, very low-density lipoprotein; LDL, low-density lipoprotein. ^$^ Fisher’s exact test.

**Table 2:** Correlation analysis between collagen genes and nuclear receptor coactivators.

Genes	R	95% CI	*p*-Value
*COL1A1* vs. *SRC-1*	0.436	0.221–0.611	<0.0005
*COL1A1* vs. *SRC-2*	0.437	0.217–0.615	<0.0005
*COL1A1* vs. *SRC-3*	0.501	0.300–0.659	<0.0005
*COL3A1* vs. *SRC-1*	0.553	0.367–0.696	<0.0005
*COL3A1* vs. *SRC-2*	0.495	0.291–0.656	<0.0005
*COL3A1* vs. *SRC-3*	0.570	0.390–0.708	<0.0005

R, correlation coefficient; CI, confidence interval.

## Data Availability

The data presented in this study are available on request from the corresponding author due to ethical reasons.

## Supplementary Materials

The following supporting information can be downloaded at: https://www.mdpi.com/article/10.3390/biomedicines13122896/s1, Figure S1: Representative image of the electrophoretic profile of the RNA samples on agarose gel; Table S1: Clinical Characteristics of the Study Cohort: Age and PSA Distribution; Table S2: TaqMan™ Assay tables were used in the study; Figure S2: Expression of *p300* in the BPH group; Figure S3: In silico analysis using the GTEx dataset showing expression levels of *COL1A1*, *COL3A1*, *EP300*, *KAT2B (PCAF)*, *NCOA1 (SRC-1)*, *NCOA2 (SRC-2)*, and *NCOA3 (SRC-3)* in BPH samples across the four Hardy Scale categories; Figure S4: Gene expression across age brackets in an in-silico analysis using the GTEx dataset. Boxplots showing expression levels of *COL1A1*, *COL3A1*, *EP300*, *KAT2B (PCAF)*, *NCOA1 (SRC-1)*, *NCOA2 (SRC-2)*, and *NCOA3 (SRC-3)* in BPH samples across different age brackets (20–29, 30–39, 40–49, and 50–59); Figure S5: Gene expression across age brackets in an in-silico analysis using the GTEx dataset. Boxplots showing expression levels of *COL1A1*, *COL3A1*, *EP300*, *KAT2B (PCAF)*, *NCOA1 (SRC-1)*, *NCOA2 (SRC-2)*, and *NCOA3 (SRC-3)* in BPH samples across different age brackets (60–69 and 70–79).
