# Supplementary material for: Molecular Profile and Clinical Associations of Androgen Receptor Coactivators and Structural Genes in Benign Prostatic Hyperplasia and Metabolic Syndrome
Source: Biomedicines. 2025 Nov 27;13(12):2896. doi: 10.3390/biomedicines13122896 (PMC12730403; doi:10.3390/biomedicines13122896)

Supplementary Figure S1. Representative image of the electrophoretic profile of the RNA samples on agarose gel.

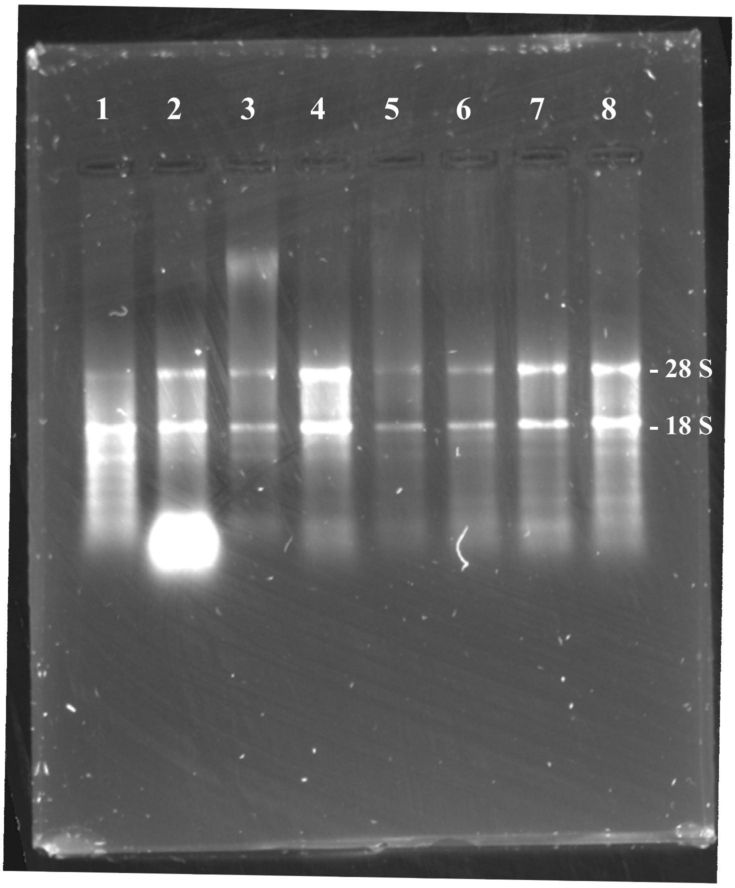

Supplementary Table S1. Clinical Characteristics of the Study Cohort: Age and PSA Distribution.

**Age group**	**Control (n, %)**	**BPH (n, %)**	**BPH (n, %)**	**p-value**
≤ 65	4 (80.00%)	27 (35.53%)	31 (38.27%)	0.070
> 65	1 (20.00%)	49 (64.47%)	50 (61.73%)	
Total	5 (100%)	76 (100%)	81 (100%)	
PSA (ng/mL)	**Control (n, %)**	**BPH (n, %)**	**Total (n, %)**	**p-value**
< 10	5 (100%)	50 (81.97%)	55 (83.33%)	0.580
> 10	0 (0%)	11 (18.03%)	11 (16.67%)	
Total	5 (100%)	61 (100%)	66 (100%)	

Supplementary Table S2. TaqMan™ Assay tables were used in the study.

Genes	TaqMan	Company
SRC-1	Hs00186661_m1	Applied Biosystems
SRC-2	Hs00896109_m1	Applied Biosystems
SRC-3	Hs00180722_m1	Applied Biosystems
PCAF	Hs00918445_g1	Applied Biosystems
p300	Hs00914223_m1	Applied Biosystems
B2M	Hs00187842_m1	Applied Biosystems

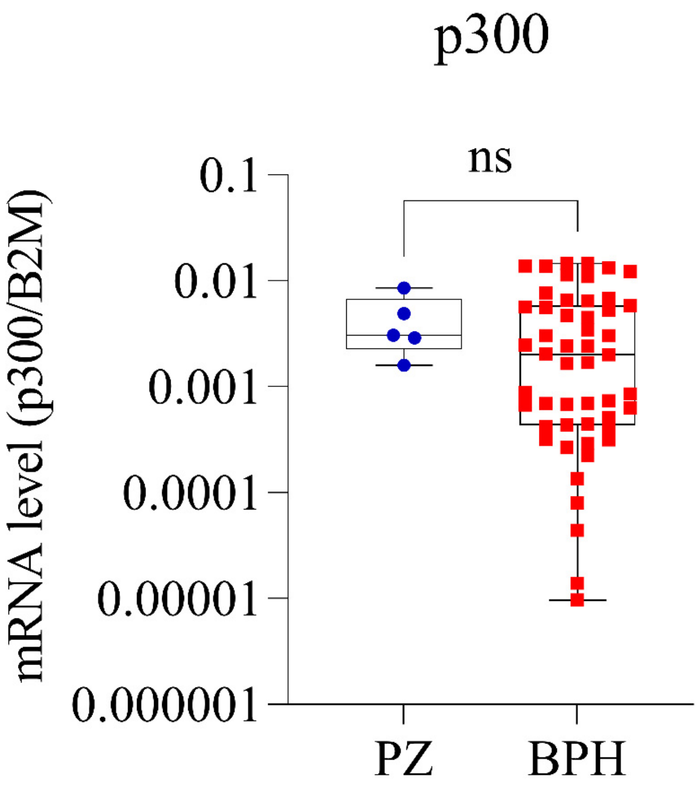

Supplementary Figure S2. Expression of *p300* in the BPH group.

Supplementary Figure S3. In silico analysis using the GTEx dataset showing expression levels of *COL1A1, COL3A1, EP300, KAT2B (PCAF), NCOA1 (SRC-1), NCOA2 (SRC-2),* and *NCOA3 (SRC-3)* in BPH samples across the four Hardy Scale categories.

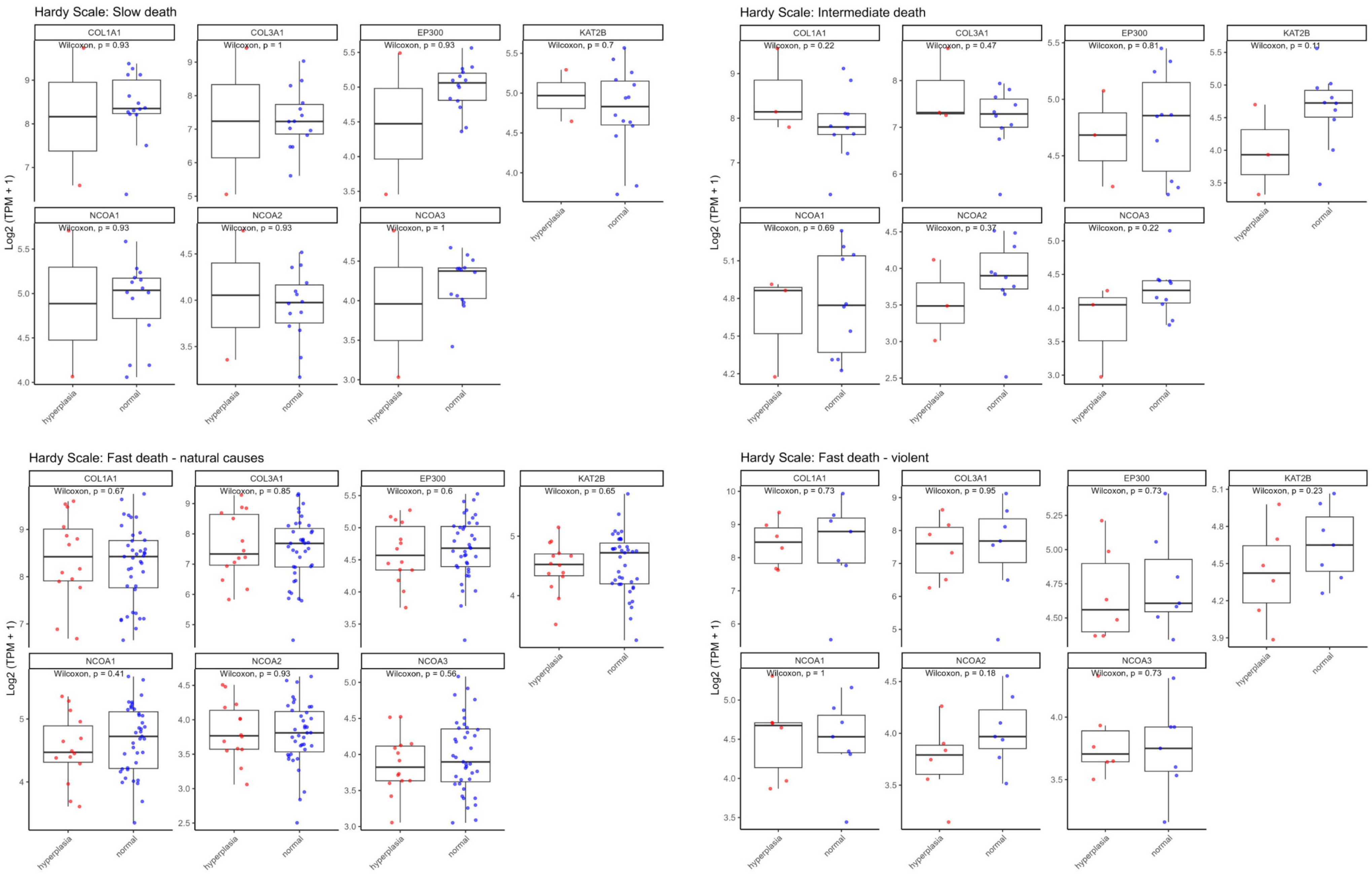

Supplementary Figure S4. Gene expression across age brackets in an in-silico analysis using the GTEx dataset. Boxplots showing expression levels of *COL1A1, COL3A1, EP300, KAT2B (PCAF), NCOA1 (SRC-1), NCOA2 (SRC-2),* and *NCOA3 (SRC-3)* in BPH samples across different age brackets (20–29, 30–39, 40–49, and 50–59).

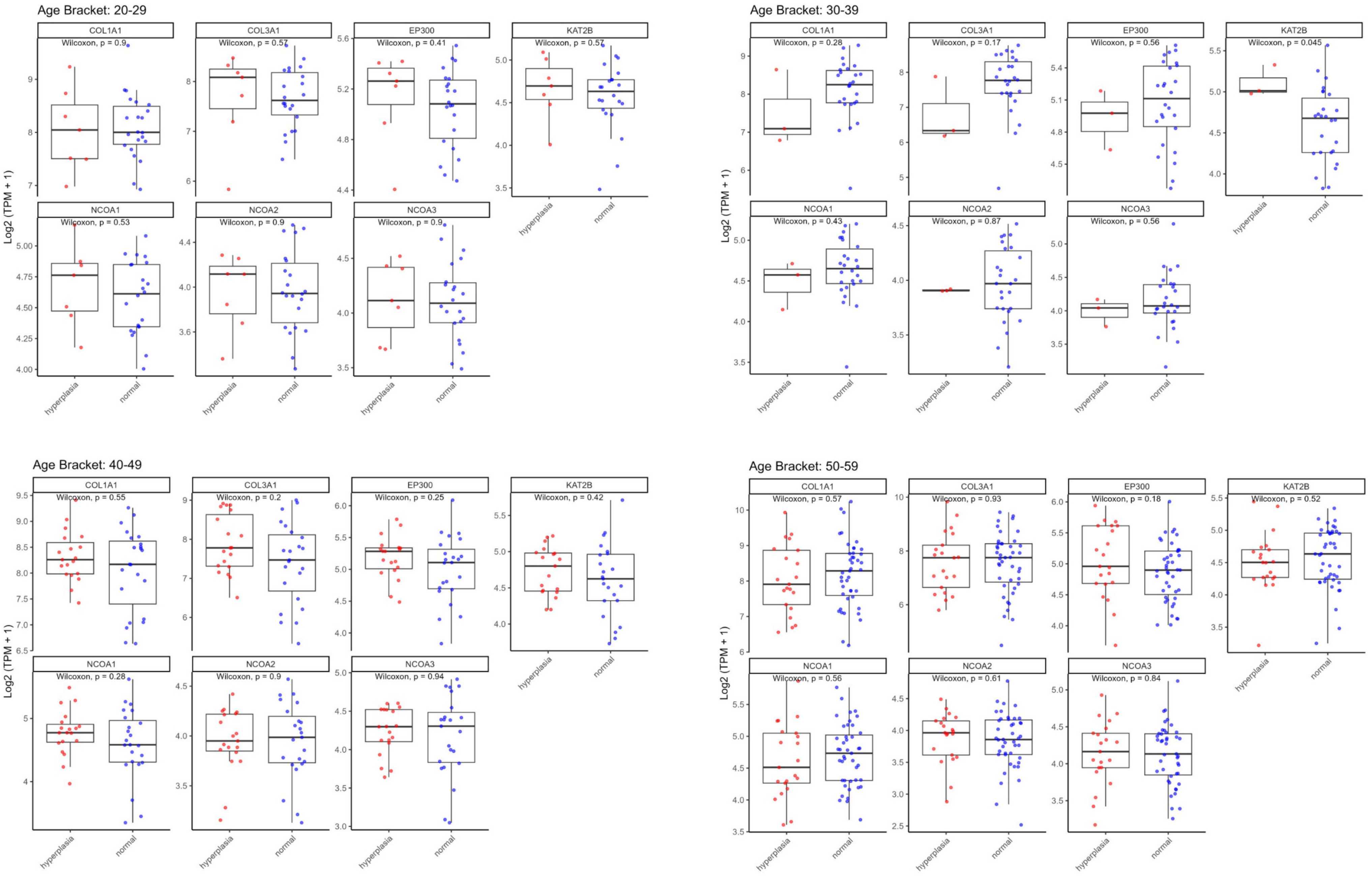

Supplementary Figure S5. Gene expression across age brackets in an in-silico analysis using the GTEx dataset. Boxplots showing expression levels of *COL1A1, COL3A1, EP300, KAT2B (PCAF), NCOA1 (SRC-1), NCOA2 (SRC-2),* and *NCOA3 (SRC-3)* in BPH samples across different age brackets (60–69 and 70–79).

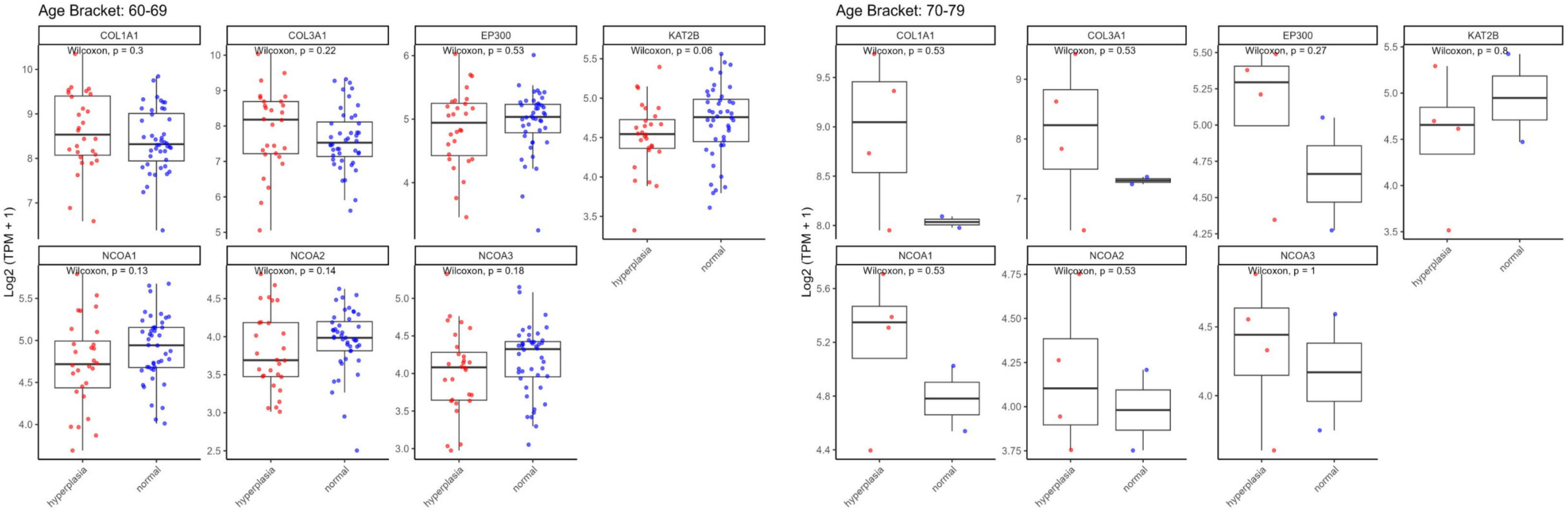

Supplement: Supplementary file 1 [file biomedicines-13-02896-s001.zip › Supplementary Material_proof revised_ID 3961919.pdf]
